# Supplementary figures and images for: PARP10 is critical for stress granule initiation
Source: Life Sci Alliance. 2025 Oct 9;8(12):e202403026. doi: 10.26508/lsa.202403026 (PMC12511761; doi:10.26508/lsa.202403026)

Figure 1

A

GFP-PARG

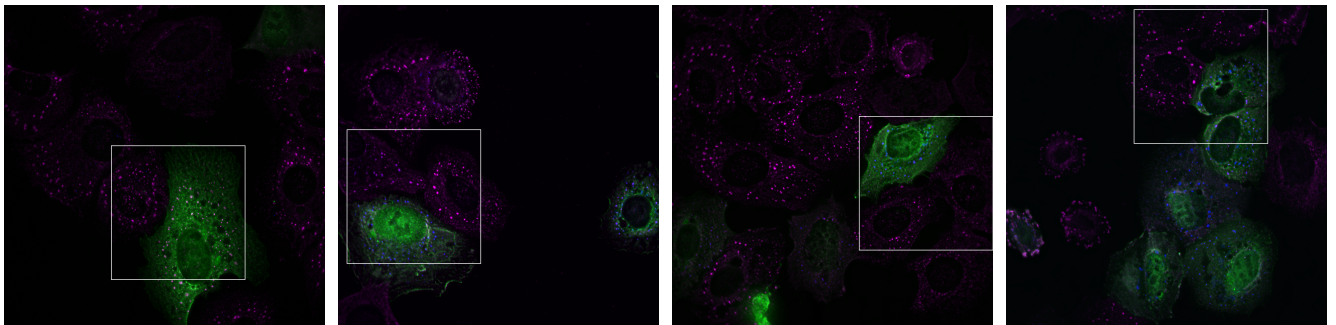

FLAG-nsP3

FLAG-nsP3

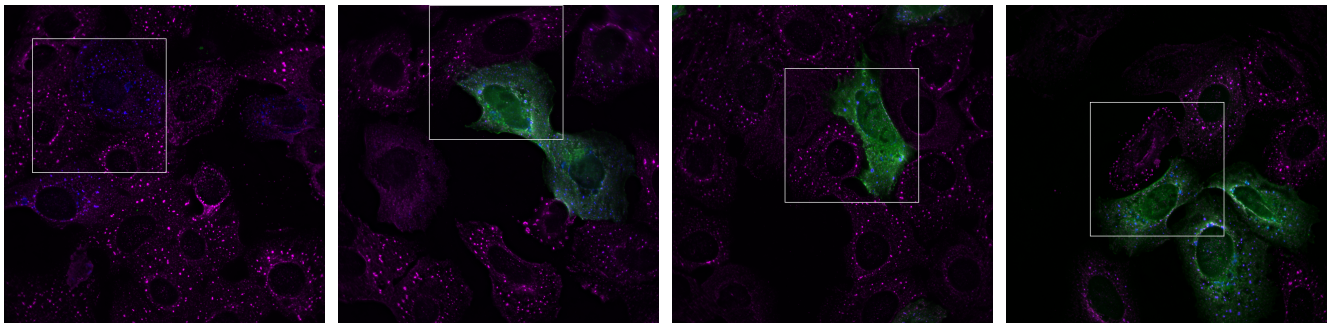

GFP-PARG

C

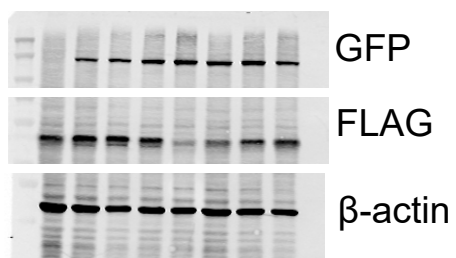

E

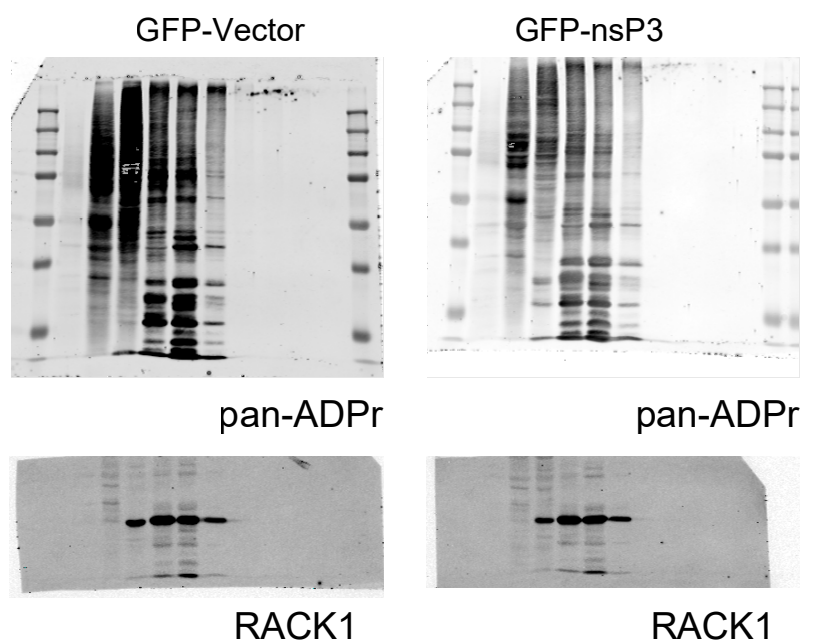

D

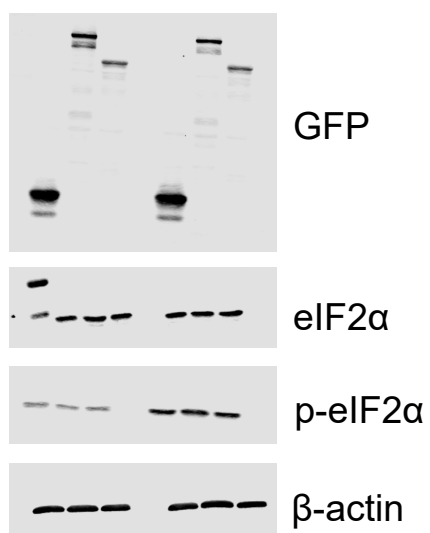

Supplement: Supplementary file 1 [file LSA-2024-03026_SdataF1.pdf]

Supplementary Figure 1

B

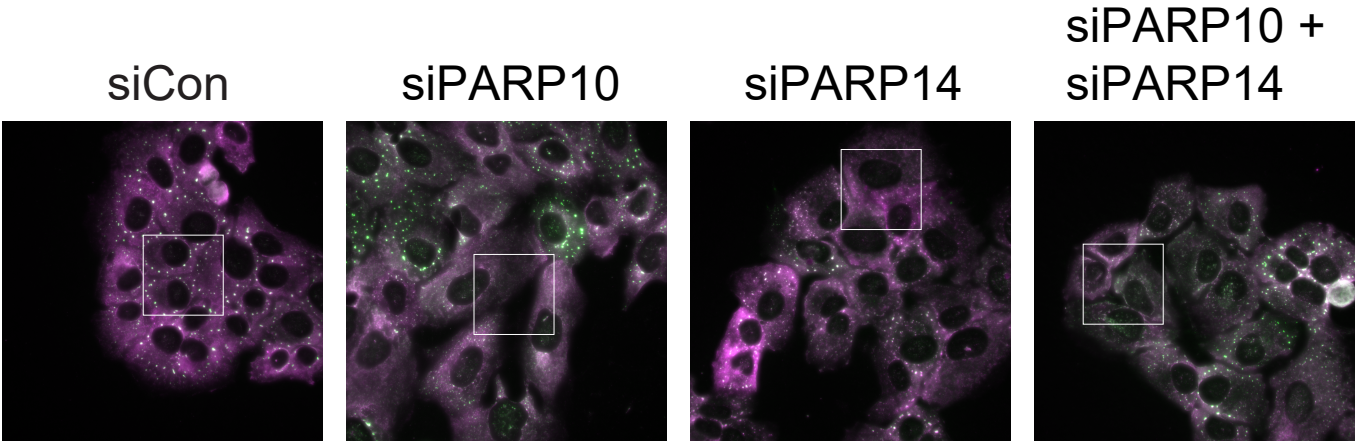

D

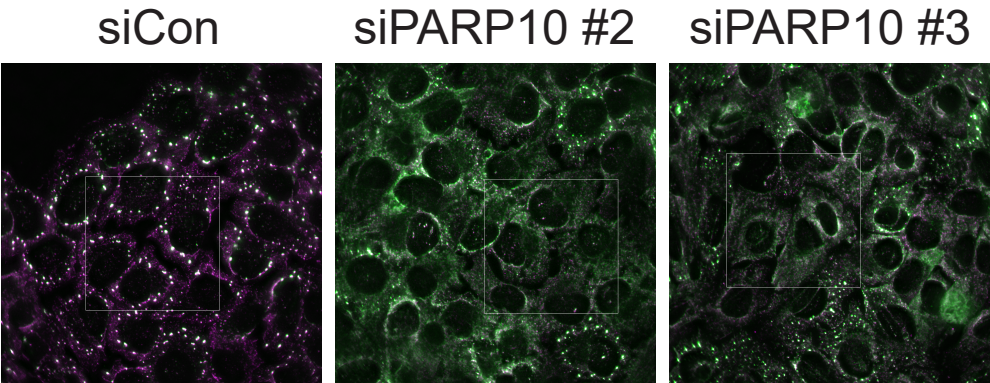

F

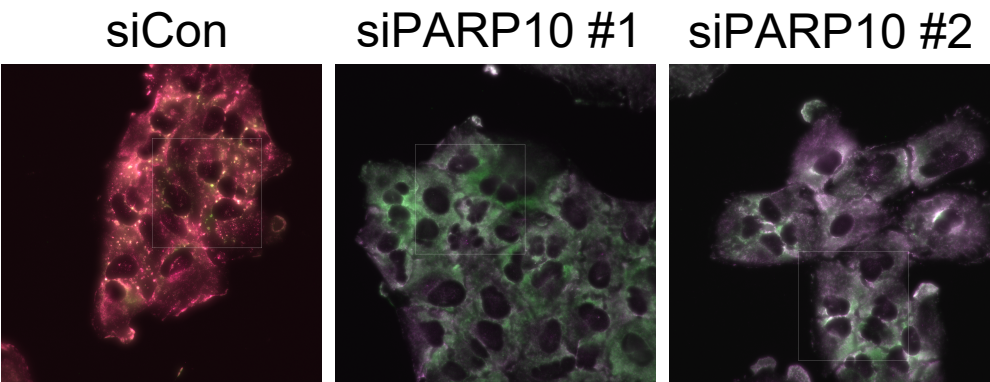

Supplement: Supplementary file 3 [file LSA-2024-03026_SdataFS1.pdf]

Supplementary Figure 2

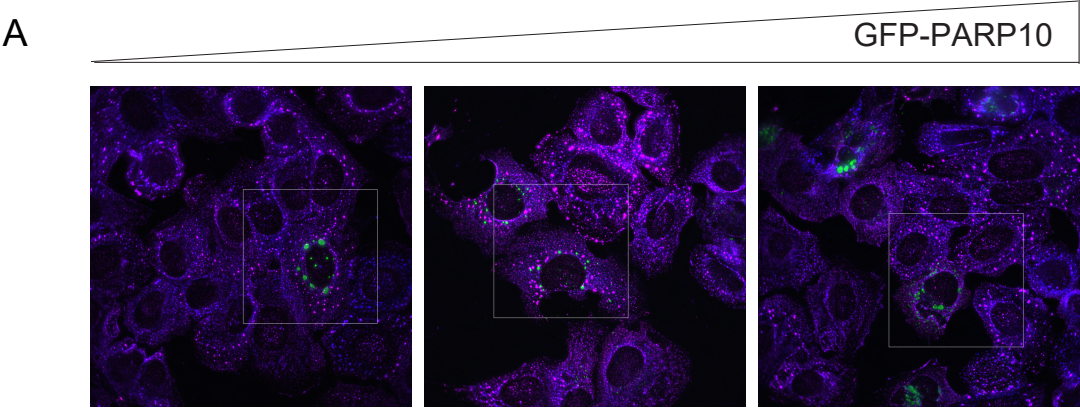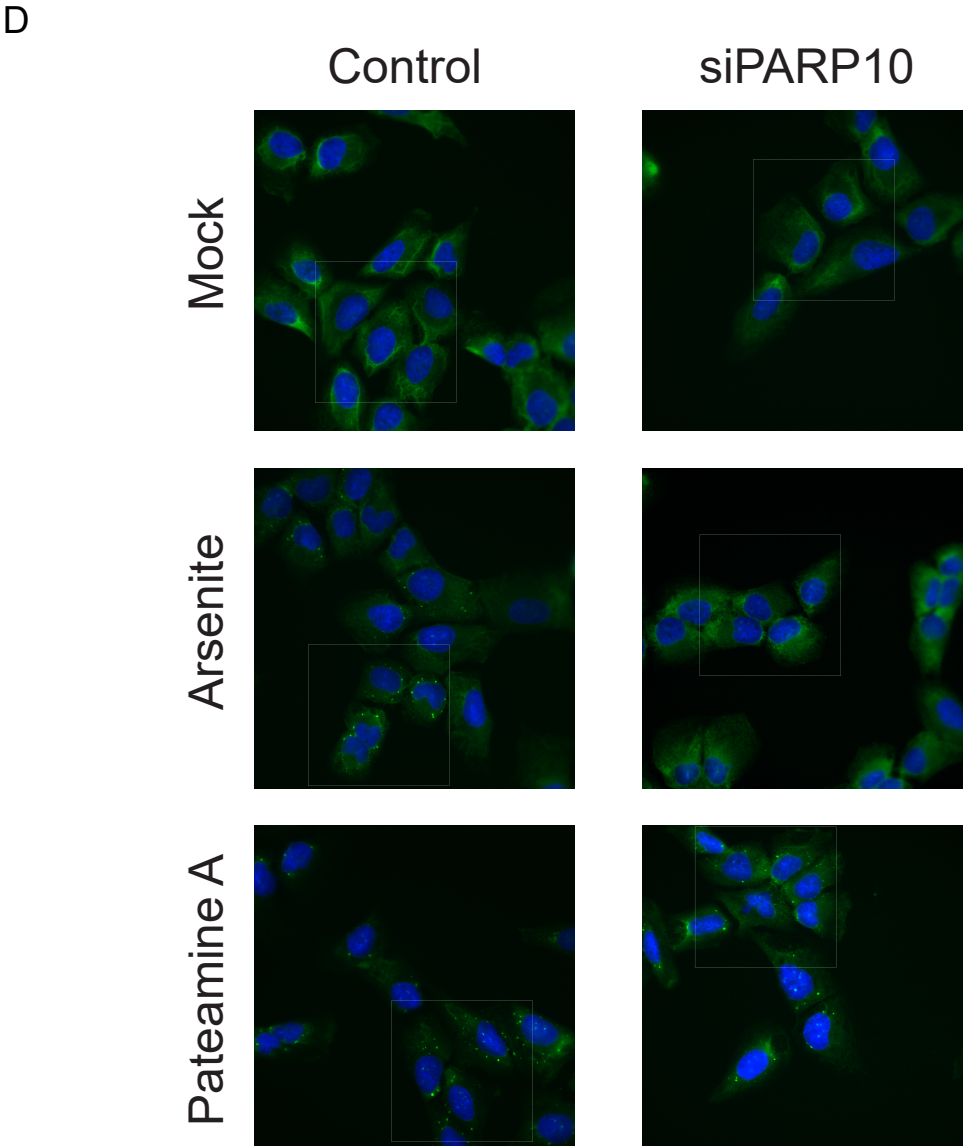

Supplement: Supplementary file 4 [file LSA-2024-03026_SdataFS2.pdf]

Figure 3

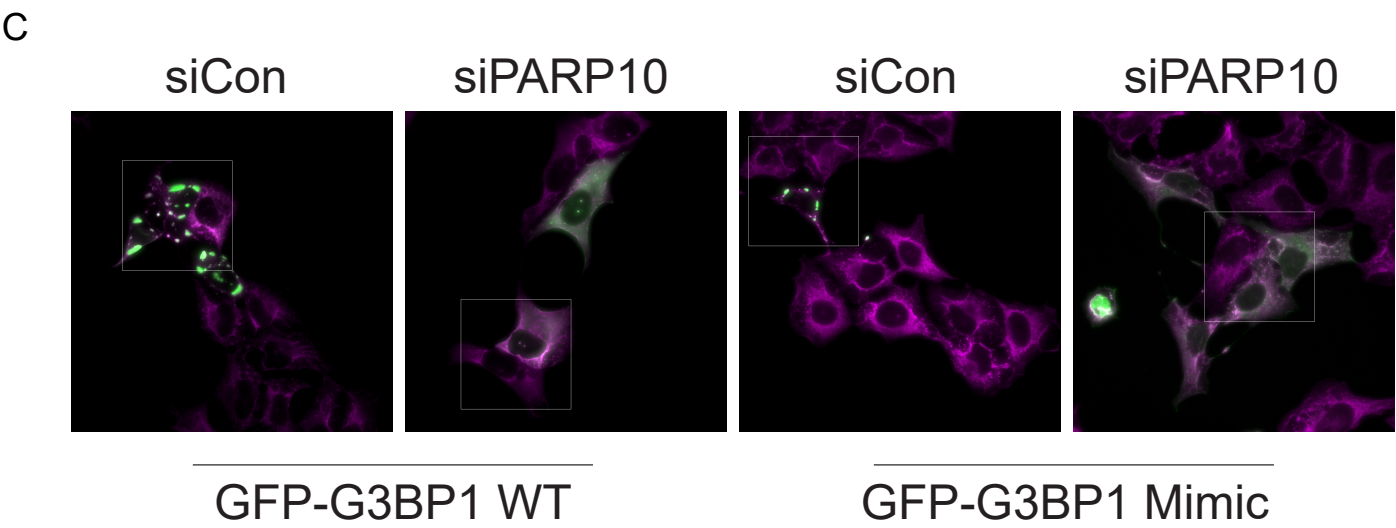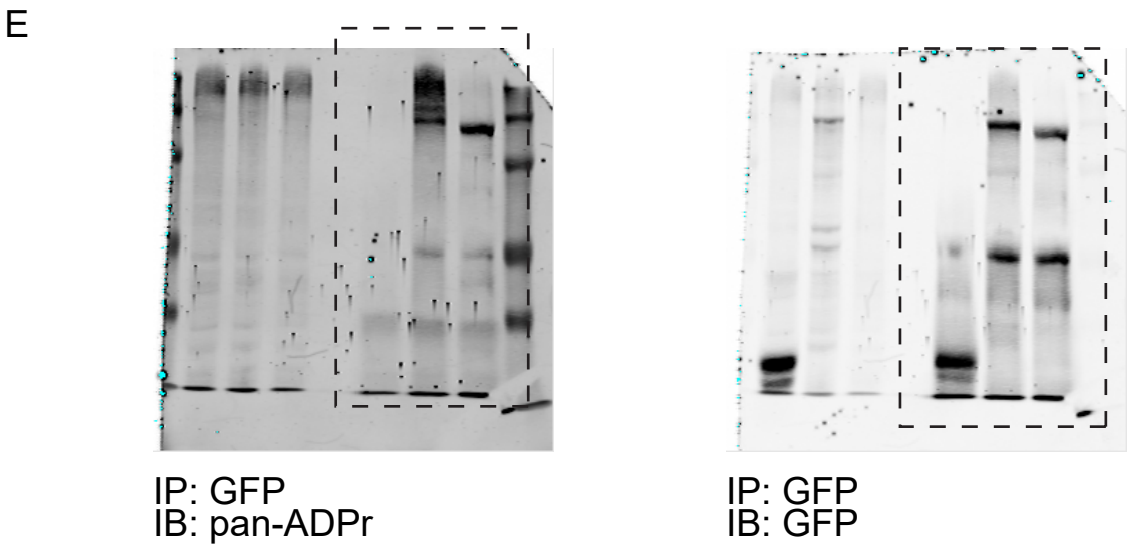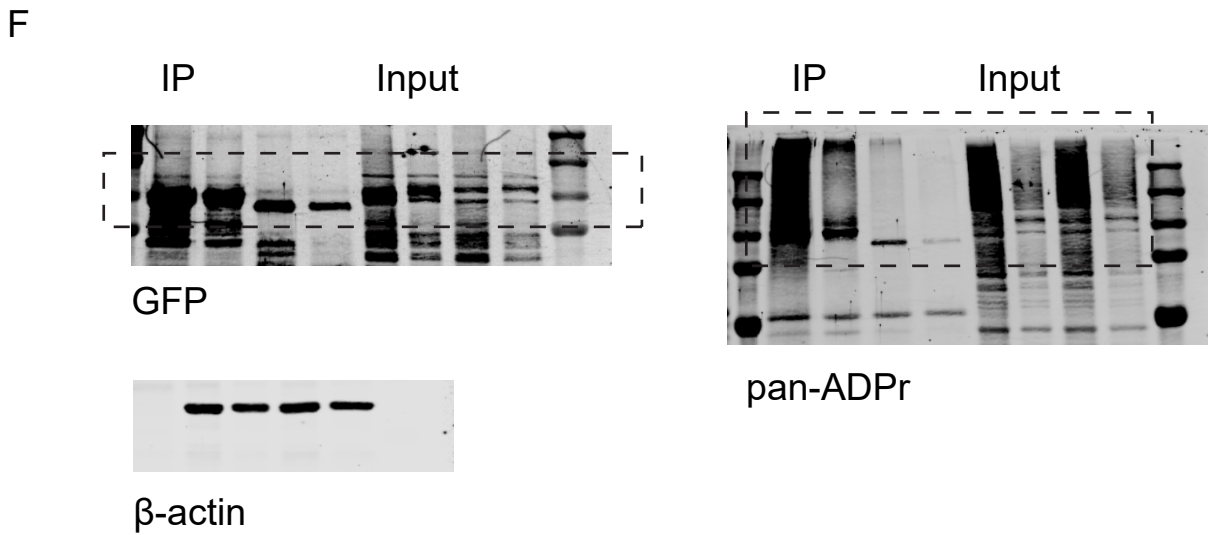

Supplement: Supplementary file 6 [file LSA-2024-03026_SdataF3.pdf]

Figure 4

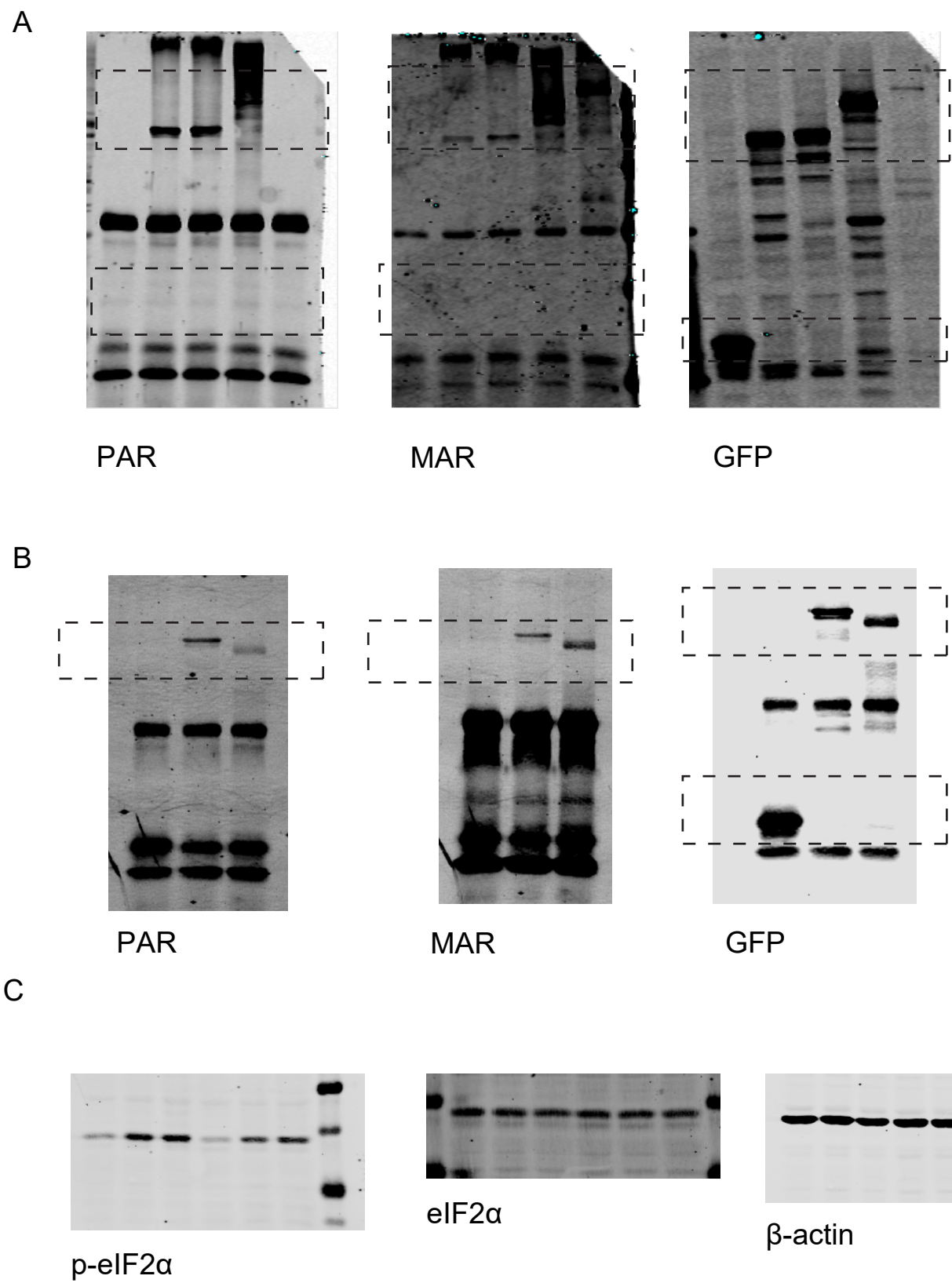

Figure 4

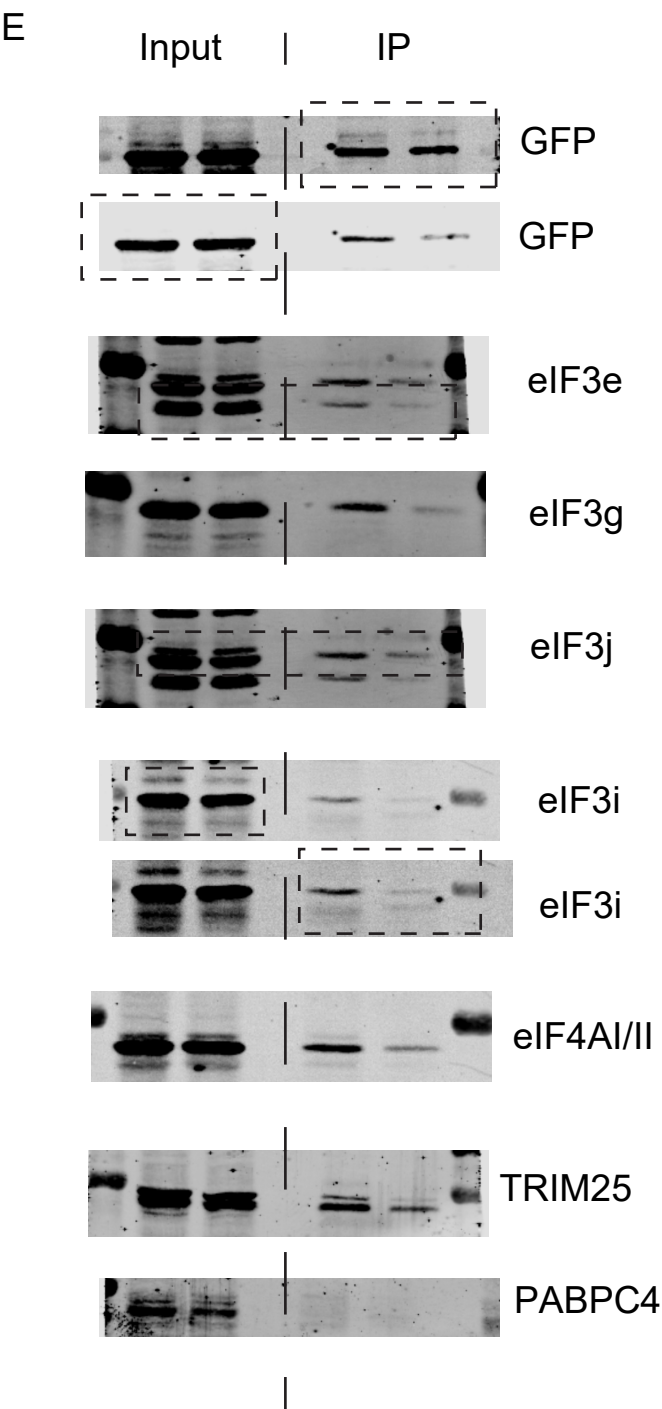

Supplement: Supplementary file 7 [file LSA-2024-03026_SdataF4.pdf]
