# Supplementary material for: PARP10 is critical for stress granule initiation
Source: Life Sci Alliance. 2025 Oct 9;8(12):e202403026. doi: 10.26508/lsa.202403026 (PMC12511761; doi:10.26508/lsa.202403026)

Figure 2

A

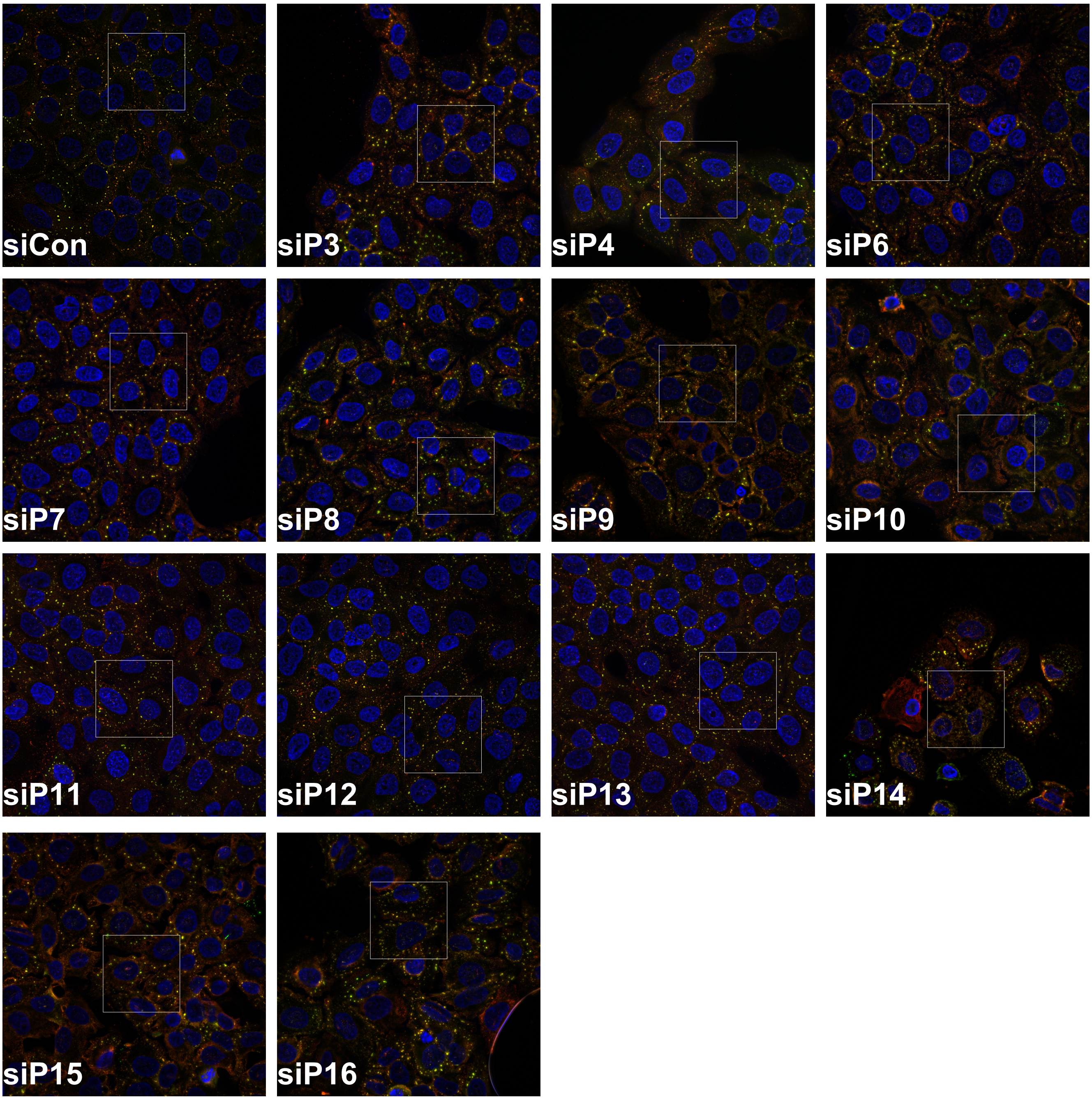
siCon
siP3
siP4
siP6
siP7
siP8
siP9
siP10
siP11
siP12
siP13
siP14
siP15
siP16

Figure 2

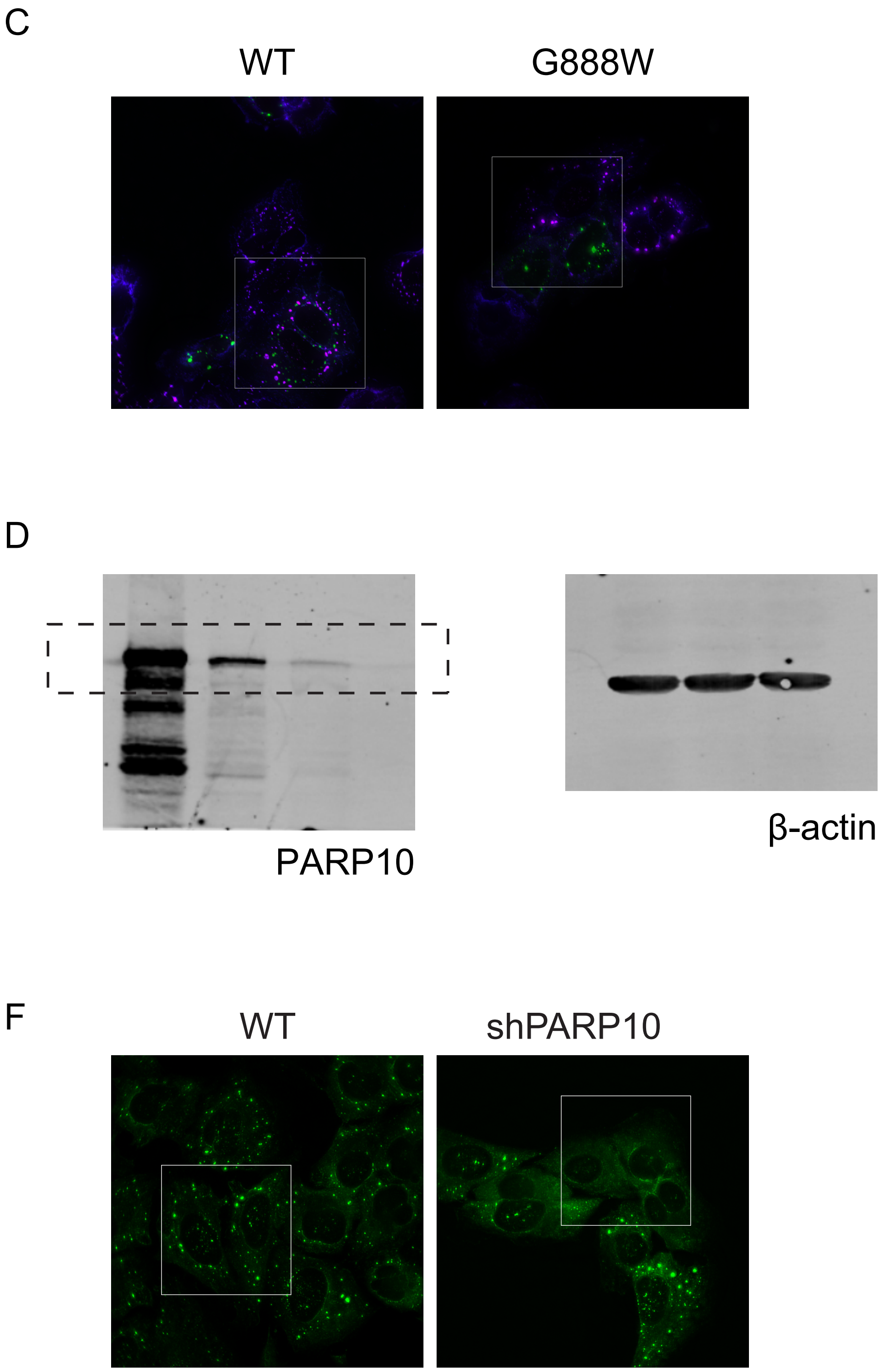

Supplement: Supplementary file 2 [file LSA-2024-03026_SdataF2.pdf]
